# 4-Hy­droxy-1-methyl-3-phenyl­quinolin-2(1*H*)-one

**DOI:** 10.1107/S1600536813000226

**Published:** 2013-01-12

**Authors:** Stanislav Kafka, Andrej Pevec, Karel Proisl, Roman Kimmel, Janez Košmrlj

**Affiliations:** a Department of Chemistry, Faculty of Technology, Tomas Bata University in Zlin, Zlin 76272, Czech Republic; b Faculty of Chemistry and Chemical Technology, University of Ljubljana, SI-1000 Ljubljana, Slovenia

## Abstract

In the title compound, C_16_H_13_NO_2_, the quinoline system is approximately planar with a maximum deviation from the least-squares plane of 0.059 (1) Å for the N atom. The phenyl ring is rotated by 62.16 (4)° with respect to the plane of the quinoline system. In the crystal, O—H⋯O hydrogen bonds link mol­ecules into infinite chains running along the *b-*axis direction.

## Related literature

For the preparation of the title compound and other 4-hy­droxy­quinolin-2-ones, see: Baumgarten & Kärgel (1927 ▶); Lange *et al.*, (2001 ▶); Martensson & Nilsson (1960 ▶); Bezuglyi *et al.* (1992 ▶). For synthetic utilization of the title compound, see: Kafka *et al.* (2002 ▶); Klásek *et al.* (2002 ▶).
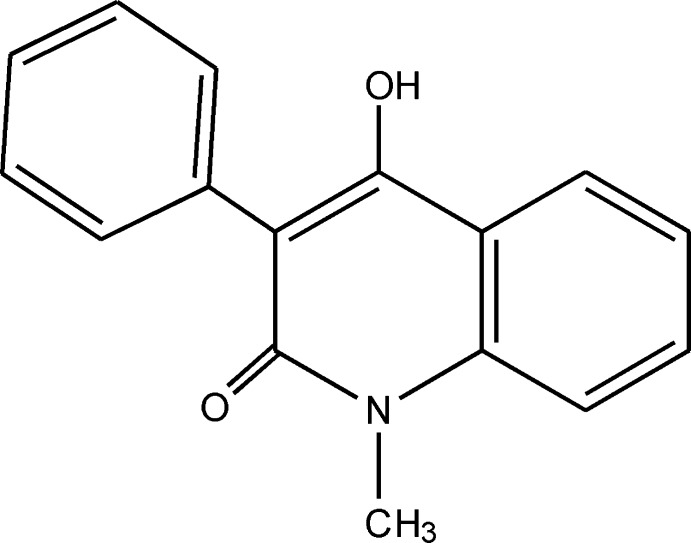


## Experimental

### 

#### Crystal data

C_16_H_13_NO_2_
*M* *_r_* = 251.27
Monoclinic, 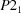

*a* = 6.1787 (2) Å
*b* = 8.2696 (2) Å
*c* = 12.3665 (4) Å
β = 101.632 (2)°
*V* = 618.89 (3) Å^3^
*Z* = 2
Mo *K*α radiation
μ = 0.09 mm^−1^
*T* = 293 K
0.50 × 0.25 × 0.10 mm

#### Data collection

Nonius KappaCCD area-detector diffractometer
Absorption correction: multi-scan (*SCALEPACK*; Otwinowski & Minor, 1997 ▶) *T*_min_ = 0.957, *T*_max_ = 0.991
2580 measured reflections
1479 independent reflections
1235 reflections with *I* > 2σ(*I*)
*R*_int_ = 0.017

#### Refinement

*R*[*F* ^2^ > 2σ(*F* ^2^)] = 0.039
*wR*(*F* ^2^) = 0.102
*S* = 1.02
1479 reflections
174 parameters
1 restraint
H-atom parameters constrained
Δρ_max_ = 0.13 e Å^−3^
Δρ_min_ = −0.16 e Å^−3^

### 

Data collection: *COLLECT* (Nonius, 1998 ▶); cell refinement: *DENZO* and *SCALEPACK* (Otwinowski & Minor, 1997 ▶); data reduction: *DENZO* and *SCALEPACK*; program(s) used to solve structure: *SHELXS97* (Sheldrick, 2008 ▶); program(s) used to refine structure: *SHELXL97* (Sheldrick, 2008 ▶); molecular graphics: *PLATON* (Spek, 2009 ▶) and *DIAMOND* (Brandenburg, 1999 ▶); software used to prepare material for publication: *WinGX* (Farrugia, 2012 ▶).

## Supplementary Material

Crystal structure: contains datablock(s) I, global. DOI: 10.1107/S1600536813000226/fy2079sup1.cif

Structure factors: contains datablock(s) I. DOI: 10.1107/S1600536813000226/fy2079Isup2.hkl

Supplementary material file. DOI: 10.1107/S1600536813000226/fy2079Isup3.cml

Additional supplementary materials:  crystallographic information; 3D view; checkCIF report

## Figures and Tables

**Table 1:** Hydrogen-bond geometry (Å, °)

*D*—H⋯*A*	*D*—H	H⋯*A*	*D*⋯*A*	*D*—H⋯*A*
O2—H2*O*⋯O1^i^	0.82	1.89	2.655 (2)	156

Symmetry code: (i) 
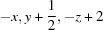
.

**Fig. 1.**
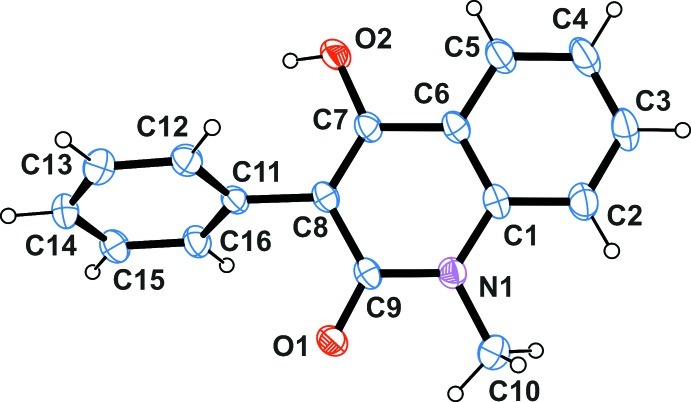
A view of the title compound showing the atom-numbering scheme. Displacement ellipsoids are drawn at the 30% probability level.

**Fig. 2.**
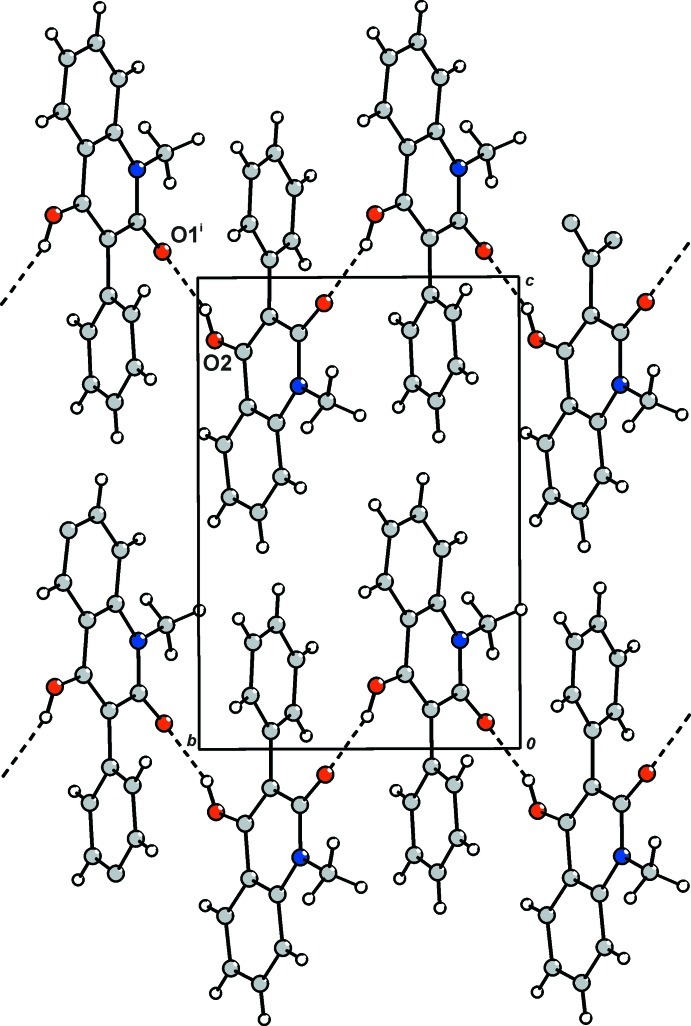
The packing of (I), with the O—H···O hydrogen bonds. [Symmetry code: (i) -x, y + 1/2, -z + 2.]

## supplementary crystallographic information

### Comment

The title compound, (I) (Fig. 1), was first prepared by the thermal condensation of diethyl phenylmalonate with *N*-methylaniline (Baumgarten & Kärgel 1927). This reaction, performed with various malonates and anilines, is still the most widely used general method for the preparation of 4-hydroxyquinolin-2-ones, including compound I. The performance of the reaction under microwave irradiation was described by Lange *et al.* (2001). Among other approaches to the preparation of compound I and other 4-hydroxyquinoline-2-diones, intramolecular condensations of 2-acylaminobenzoates could be particularly feasible (Martensson & Nilsson, 1960; Bezuglyi *et al.*, 1992). Recently, compound I was utilized for the preparation of the corresponding 3-bromo- and 3-chloro-1-methyl-3-phenylquinoline-2,4(1*H*,3*H*)-diones, from which other compounds were prepared by nucleophilic substitution of the halogen atom (Kafka *et al.*, 2002; Klásek *et al.*, 2002).

In the crystal structure of the title compound (I) (Fig. 2) 4-hydroxy-1-methyl-3-phenylquinolin-2(1*H*)-one molecules are connected by intermolecular O—H···O hydrogen bonds between the hydroxyl and carbonyl groups (Table 1). These connections form linear chains along the *b*-axis in the crystal structure.

### Experimental

Title compound was prepared according to a modified procedure published by Baumgarten & Kärgel (1927). A mixture of *N*-methylaniline (10.7 g, 100 mmol) and diethyl phenylmalonate (24.8 g, 105 mmol) was gradually heated in a Wood's metal bath at 200–290 °C for 4.5 h (until the distillation of ethanol stopped; reached 8.57 g, *i.e.* 93% of theoretical mass of distillate). The hot reaction mixture was poured into a mortar, crushed after cooling and dissolved in the mixture of aqueous sodium hydroxide solution (0.5 *M*, 300 ml) and toluene (50 ml). The aqueous phase was separated, washed with toluene, shortly stirred with active carbon, filtered and acidified by addition of 10% hydrochloric acid to Kongo red. The precipitated white solid was filtered off, washed with water and air dried affording 23.4 g (93% of theory) of crude product, m. p. 223–225 C. Crystallization of the crude product from ethanol afforded 20.1 g (80% of theoretical yield) of the title compound (I), m. p. 222–226 °C. In the literature (Martensson & Nilsson, 1960), the same m. p. is given.

### Refinement

All H atoms were included in the model at geometrically calculated positions and refined using a riding model, with C—H bond lengths constrained to 0.93 Å (aromatic CH), 0.96 Å (methyl CH_3_), and O—H = 0.82 Å, and with *U*_iso_(H) values of 1.2*U*_eq_(C) [for aromatic CH] or 1.5*U*_eq_(C) [for OH and methyl groups]. In the absence of significant anomalous scattering, the Flack parameter could not be determined reliably. Therefore Friedel-pairs were merged prior to the final refinement cycle. 16 low angle reflections were dropped by the integration routines because of detector saturation.

### Crystal data

**Table:**

C_16_H_13_NO_2_	*F*(000) = 264
*M**_r_* = 251.27	*D*_x_ = 1.348 Mg m^−^^3^
Monoclinic, *P*2_1_	Melting point = 495–499 K
Hall symbol: P 2yb	Mo *K*α radiation, λ = 0.71073 Å
*a* = 6.1787 (2) Å	Cell parameters from 1471 reflections
*b* = 8.2696 (2) Å	θ = 1.0–27.5°
*c* = 12.3665 (4) Å	µ = 0.09 mm^−^^1^
β = 101.632 (2)°	*T* = 293 K
*V* = 618.89 (3) Å^3^	Prism, colorless
*Z* = 2	0.50 × 0.25 × 0.10 mm

### Data collection

**Table:**

Nonius KappaCCD area-detector diffractometer	1479 independent reflections
Radiation source: fine-focus sealed tube	1235 reflections with *I* > 2σ(*I*)
Graphite monochromator	*R*_int_ = 0.017
φ scans + ω scans	θ_max_ = 27.4°, θ_min_ = 5.5°
Absorption correction: multi-scan (*SCALEPACK*; Otwinowski & Minor, 1997)	*h* = −7→7
*T*_min_ = 0.957, *T*_max_ = 0.991	*k* = −10→8
2580 measured reflections	*l* = −16→15

### Refinement

**Table:**

Refinement on *F*^2^	Primary atom site location: structure-invariant direct methods
Least-squares matrix: full	Secondary atom site location: difference Fourier map
*R*[*F*^2^ > 2σ(*F*^2^)] = 0.039	Hydrogen site location: inferred from neighbouring sites
*wR*(*F*^2^) = 0.102	H-atom parameters constrained
*S* = 1.02	*w* = 1/[σ^2^(*F*_o_^2^) + (0.0575*P*)^2^ + 0.0786*P*] where *P* = (*F*_o_^2^ + 2*F*_c_^2^)/3
1479 reflections	(Δ/σ)_max_ = 0.0001
174 parameters	Δρ_max_ = 0.13 e Å^−^^3^
1 restraint	Δρ_min_ = −0.16 e Å^−^^3^

### Special details

**Table:**

Experimental. 216 frames in 4 sets of φ scans + ω scans. Rotation/frame = 2.0 °. Crystal-detector distance = 31 mm. Measuring time = 200 s/°.
Geometry. All s.u.'s (except the s.u. in the dihedral angle between two l.s. planes) are estimated using the full covariance matrix. The cell s.u.'s are taken into account individually in the estimation of s.u.'s in distances, angles and torsion angles; correlations between s.u.'s in cell parameters are only used when they are defined by crystal symmetry. An approximate (isotropic) treatment of cell s.u.'s is used for estimating s.u.'s involving l.s. planes.
Refinement. Refinement of *F*^2^ against ALL reflections. The weighted *R*-factor *wR* and goodness of fit *S* are based on *F*^2^, conventional *R*-factors *R* are based on *F*, with *F* set to zero for negative *F*^2^. The threshold expression of *F*^2^ > 2σ(*F*^2^) is used only for calculating *R*-factors(gt) *etc*. and is not relevant to the choice of reflections for refinement. *R*-factors based on *F*^2^ are statistically about twice as large as those based on *F*, and *R*- factors based on ALL data will be even larger.

### Fractional atomic coordinates and isotropic or equivalent isotropic displacement parameters (Å^2^)

**Table:**

	*x*	*y*	*z*	*U*_iso_*/*U*_eq_	
N1	−0.3506 (3)	0.6870 (2)	0.76995 (14)	0.0421 (5)	
O1	−0.3484 (2)	0.6084 (2)	0.94498 (12)	0.0461 (4)	
O2	0.2230 (3)	0.9476 (3)	0.86700 (12)	0.0527 (5)	
H2O	0.2433	0.9751	0.9320	0.079*	
C1	−0.2491 (4)	0.7574 (3)	0.69150 (17)	0.0408 (5)	
C2	−0.3367 (5)	0.7420 (4)	0.57760 (19)	0.0563 (7)	
H2	−0.4659	0.6833	0.5536	0.068*	
C3	−0.2321 (5)	0.8131 (4)	0.50211 (19)	0.0640 (8)	
H3	−0.2903	0.8004	0.4272	0.077*	
C4	−0.0430 (6)	0.9027 (4)	0.5350 (2)	0.0631 (7)	
H4	0.0251	0.9512	0.4827	0.076*	
C5	0.0453 (5)	0.9202 (3)	0.64626 (19)	0.0531 (6)	
H5	0.1720	0.9821	0.6688	0.064*	
C6	−0.0539 (4)	0.8458 (3)	0.72531 (17)	0.0408 (5)	
C7	0.0393 (4)	0.8579 (3)	0.84224 (17)	0.0392 (5)	
C8	−0.0553 (4)	0.7782 (3)	0.91735 (16)	0.0374 (5)	
C9	−0.2559 (3)	0.6871 (3)	0.88098 (16)	0.0374 (5)	
C10	−0.5599 (4)	0.6003 (4)	0.7363 (2)	0.0558 (6)	
H10A	−0.5330	0.4973	0.7057	0.084*	
H10B	−0.6579	0.6625	0.6817	0.084*	
H10C	−0.6261	0.5843	0.7994	0.084*	
C11	0.0412 (3)	0.7767 (3)	1.03788 (15)	0.0374 (5)	
C12	0.2473 (4)	0.7070 (3)	1.07702 (19)	0.0456 (5)	
H12	0.3288	0.6669	1.0274	0.055*	
C13	0.3319 (4)	0.6971 (4)	1.1895 (2)	0.0537 (6)	
H13	0.4682	0.6483	1.2151	0.064*	
C14	0.2142 (5)	0.7594 (4)	1.26318 (19)	0.0568 (7)	
H14	0.2709	0.7523	1.3386	0.068*	
C15	0.0133 (4)	0.8321 (4)	1.22571 (19)	0.0535 (6)	
H15	−0.0640	0.8764	1.2758	0.064*	
C16	−0.0749 (4)	0.8396 (3)	1.11342 (18)	0.0460 (6)	
H16	−0.2124	0.8871	1.0886	0.055*	

### Atomic displacement parameters (Å^2^)

**Table:**

	*U*^11^	*U*^22^	*U*^33^	*U*^12^	*U*^13^	*U*^23^
N1	0.0427 (10)	0.0467 (10)	0.0362 (9)	0.0025 (9)	0.0062 (7)	0.0040 (9)
O1	0.0438 (8)	0.0554 (10)	0.0408 (8)	0.0007 (8)	0.0124 (6)	0.0101 (7)
O2	0.0653 (10)	0.0598 (10)	0.0366 (8)	−0.0198 (9)	0.0191 (8)	−0.0084 (8)
C1	0.0524 (12)	0.0392 (11)	0.0311 (10)	0.0092 (10)	0.0095 (9)	0.0020 (9)
C2	0.0630 (15)	0.0639 (17)	0.0397 (12)	0.0002 (13)	0.0047 (11)	0.0030 (12)
C3	0.087 (2)	0.0729 (19)	0.0299 (11)	0.0020 (17)	0.0058 (12)	0.0045 (12)
C4	0.0925 (19)	0.0656 (17)	0.0352 (11)	−0.0053 (16)	0.0220 (12)	0.0065 (12)
C5	0.0719 (15)	0.0535 (15)	0.0376 (11)	−0.0069 (13)	0.0201 (11)	−0.0013 (12)
C6	0.0531 (13)	0.0377 (11)	0.0339 (10)	0.0027 (10)	0.0143 (9)	−0.0009 (9)
C7	0.0472 (12)	0.0402 (12)	0.0325 (10)	−0.0009 (9)	0.0136 (9)	−0.0039 (9)
C8	0.0448 (11)	0.0383 (11)	0.0312 (10)	0.0041 (9)	0.0128 (8)	−0.0010 (9)
C9	0.0418 (11)	0.0392 (11)	0.0327 (10)	0.0083 (10)	0.0115 (8)	0.0030 (9)
C10	0.0476 (12)	0.0669 (16)	0.0497 (13)	−0.0029 (13)	0.0022 (10)	0.0035 (13)
C11	0.0432 (11)	0.0398 (11)	0.0315 (10)	−0.0029 (10)	0.0127 (9)	0.0005 (9)
C12	0.0439 (12)	0.0535 (14)	0.0410 (11)	0.0019 (11)	0.0126 (9)	−0.0017 (11)
C13	0.0470 (13)	0.0618 (15)	0.0492 (13)	0.0024 (12)	0.0019 (10)	0.0052 (13)
C14	0.0621 (15)	0.0732 (17)	0.0317 (11)	−0.0115 (14)	0.0017 (10)	0.0033 (11)
C15	0.0594 (15)	0.0693 (16)	0.0353 (11)	−0.0037 (13)	0.0177 (10)	−0.0062 (12)
C16	0.0483 (12)	0.0557 (14)	0.0354 (11)	0.0045 (11)	0.0118 (9)	−0.0011 (11)

### Geometric parameters (Å, º)

**Table:**

N1—C9	1.380 (3)	C7—C8	1.363 (3)
N1—C1	1.385 (3)	C8—C9	1.443 (3)
N1—C10	1.464 (3)	C8—C11	1.490 (3)
O1—C9	1.248 (3)	C10—H10A	0.9600
O2—C7	1.339 (3)	C10—H10B	0.9600
O2—H2O	0.8200	C10—H10C	0.9600
C1—C6	1.400 (3)	C11—C16	1.389 (3)
C1—C2	1.409 (3)	C11—C12	1.393 (3)
C2—C3	1.370 (4)	C12—C13	1.386 (3)
C2—H2	0.9300	C12—H12	0.9300
C3—C4	1.374 (4)	C13—C14	1.376 (4)
C3—H3	0.9300	C13—H13	0.9300
C4—C5	1.382 (3)	C14—C15	1.373 (4)
C4—H4	0.9300	C14—H14	0.9300
C5—C6	1.397 (3)	C15—C16	1.387 (3)
C5—H5	0.9300	C15—H15	0.9300
C6—C7	1.448 (3)	C16—H16	0.9300
			
C9—N1—C1	122.40 (19)	O1—C9—N1	118.3 (2)
C9—N1—C10	117.14 (19)	O1—C9—C8	123.29 (18)
C1—N1—C10	120.37 (18)	N1—C9—C8	118.40 (18)
C7—O2—H2O	109.5	N1—C10—H10A	109.5
N1—C1—C6	119.64 (18)	N1—C10—H10B	109.5
N1—C1—C2	121.7 (2)	H10A—C10—H10B	109.5
C6—C1—C2	118.7 (2)	N1—C10—H10C	109.5
C3—C2—C1	120.2 (2)	H10A—C10—H10C	109.5
C3—C2—H2	119.9	H10B—C10—H10C	109.5
C1—C2—H2	119.9	C16—C11—C12	118.78 (19)
C2—C3—C4	121.3 (2)	C16—C11—C8	120.87 (19)
C2—C3—H3	119.3	C12—C11—C8	120.32 (18)
C4—C3—H3	119.3	C13—C12—C11	120.4 (2)
C3—C4—C5	119.5 (2)	C13—C12—H12	119.8
C3—C4—H4	120.3	C11—C12—H12	119.8
C5—C4—H4	120.3	C14—C13—C12	120.0 (2)
C4—C5—C6	120.7 (2)	C14—C13—H13	120.0
C4—C5—H5	119.7	C12—C13—H13	120.0
C6—C5—H5	119.7	C15—C14—C13	120.2 (2)
C5—C6—C1	119.60 (19)	C15—C14—H14	119.9
C5—C6—C7	121.7 (2)	C13—C14—H14	119.9
C1—C6—C7	118.66 (19)	C14—C15—C16	120.2 (2)
O2—C7—C8	124.90 (19)	C14—C15—H15	119.9
O2—C7—C6	114.51 (18)	C16—C15—H15	119.9
C8—C7—C6	120.6 (2)	C15—C16—C11	120.3 (2)
C7—C8—C9	119.96 (18)	C15—C16—H16	119.8
C7—C8—C11	123.1 (2)	C11—C16—H16	119.8
C9—C8—C11	116.93 (18)		
			
C9—N1—C1—C6	−6.3 (3)	C6—C7—C8—C11	175.7 (2)
C10—N1—C1—C6	177.3 (2)	C1—N1—C9—O1	−173.6 (2)
C9—N1—C1—C2	173.3 (2)	C10—N1—C9—O1	2.9 (3)
C10—N1—C1—C2	−3.0 (3)	C1—N1—C9—C8	6.6 (3)
N1—C1—C2—C3	−180.0 (2)	C10—N1—C9—C8	−177.0 (2)
C6—C1—C2—C3	−0.3 (4)	C7—C8—C9—O1	178.3 (2)
C1—C2—C3—C4	−1.1 (5)	C11—C8—C9—O1	−0.4 (3)
C2—C3—C4—C5	0.7 (5)	C7—C8—C9—N1	−1.8 (3)
C3—C4—C5—C6	1.0 (5)	C11—C8—C9—N1	179.48 (19)
C4—C5—C6—C1	−2.3 (4)	C7—C8—C11—C16	118.9 (3)
C4—C5—C6—C7	178.0 (3)	C9—C8—C11—C16	−62.4 (3)
N1—C1—C6—C5	−178.4 (2)	C7—C8—C11—C12	−63.2 (3)
C2—C1—C6—C5	1.9 (3)	C9—C8—C11—C12	115.5 (2)
N1—C1—C6—C7	1.4 (3)	C16—C11—C12—C13	1.7 (4)
C2—C1—C6—C7	−178.3 (2)	C8—C11—C12—C13	−176.3 (2)
C5—C6—C7—O2	1.2 (3)	C11—C12—C13—C14	−1.4 (4)
C1—C6—C7—O2	−178.5 (2)	C12—C13—C14—C15	−0.2 (5)
C5—C6—C7—C8	−177.1 (2)	C13—C14—C15—C16	1.6 (5)
C1—C6—C7—C8	3.2 (3)	C14—C15—C16—C11	−1.3 (4)
O2—C7—C8—C9	179.0 (2)	C12—C11—C16—C15	−0.3 (4)
C6—C7—C8—C9	−2.9 (3)	C8—C11—C16—C15	177.6 (2)
O2—C7—C8—C11	−2.4 (4)		

### Hydrogen-bond geometry (Å, º)

**Table:**

*D*—H···*A*	*D*—H	H···*A*	*D*···*A*	*D*—H···*A*
O2—H2*O*···O1^i^	0.82	1.89	2.655 (2)	156

Symmetry code: (i) −*x*, *y*+1/2, −*z*+2.
